# A marginalized two-part Beta regression model for microbiome compositional data

**DOI:** 10.1371/journal.pcbi.1006329

**Published:** 2018-07-23

**Authors:** Haitao Chai, Hongmei Jiang, Lu Lin, Lei Liu

**Affiliations:** 1 Institute for Financial Studies, Shandong University, Jinan, Shandong, China; 2 Department of Preventive Medicine, Northwestern University, Chicago, Illinois, United States of America; 3 Department of Statistics, Northwestern University, Evanston, Illinois, United States of America; 4 Division of Biostatistics, Washington University in St. Louis, St. Louis, Missouri, United States of America; University of Minnesota, UNITED STATES

## Abstract

In microbiome studies, an important goal is to detect differential abundance of microbes across clinical conditions and treatment options. However, the microbiome compositional data (quantified by relative abundance) are highly skewed, bounded in [0, 1), and often have many zeros. A two-part model is commonly used to separate zeros and positive values explicitly by two submodels: a logistic model for the probability of a specie being present in Part I, and a Beta regression model for the relative abundance conditional on the presence of the specie in Part II. However, the regression coefficients in Part II cannot provide a marginal (unconditional) interpretation of covariate effects on the microbial abundance, which is of great interest in many applications. In this paper, we propose a marginalized two-part Beta regression model which captures the zero-inflation and skewness of microbiome data and also allows investigators to examine covariate effects on the marginal (unconditional) mean. We demonstrate its practical performance using simulation studies and apply the model to a real metagenomic dataset on mouse skin microbiota. We find that under the proposed marginalized model, without loss in power, the likelihood ratio test performs better in controlling the type I error than those under conventional methods.

## Author summary

Semi-continuous compositional data are typically analyzed using two-part models which separately describe the probability of zero values and the distribution of positive values. The second part of the model provides a conditional interpretation of covariate effects on the positive response. However, it is of great interest in many applications to assess the covariate effect on the marginal mean of the response. For this purpose, we propose a marginalized two-part model by reparameterizing the marginal mean in Part II. We show that the proposed marginalized two-part model outperforms conventional methods by simulation studies in terms of controlling the Type I error and maximizing the power. We apply our method to a microbiota dataset, and find consistent results with our simulation studies.

## Introduction

In recent years, metagenomics studies have been growing rapidly due to the advances of next-generation sequencing (NGS) technologies [1]. Microbiota have been known to be associated with various diseases, e.g., obesity and diabetes [2, 3], Crohn’s disease [4], bacterial vaginosis [5], and cancer [6, 7].

The microbial abundance is usually measured in read counts. However, such quantities are not directly comparable across samples due to the uneven total sequence counts of samples. Therefore, the read counts are often normalized to relative abundances which sum to 1 for all microbes in a sample [8]. Relative abundance can be characterized by a point mass at zero and a right-skewed continuous distribution with a positive support, the so-called “semi-continuous” or “zero-inflated continuous” data. The zero values indicate that certain microbes are absent in the sample, or the rare microbes are present but missed due to undersampling, while the continuous distribution with a positive support describes the levels of relative abundance among the present microbes.

The relative abundance is often described by a two-part model [9], which separates zeros and positive values explicitly by two submodels: a logistic model for the probability of the outcome being positive in Part I and a (generalized) linear regression model for the amount of the (transformed) positive value in Part II. An important issue in such two-part models is to determine the distributional form in Part II. The nonzero relative abundance data are non-normally distributed and bounded in [0, 1). Beta distribution has been used to model this outcome. A two-part Beta regression model can be thus developed [10–12]. It includes two sets of parameters, one in the logistic regression for the presence of a microbe, and the other in the Beta regression for the relative abundance conditional on the presence of the microbe. These two sets of parameters are interpreted as effects on the presence of a microbe and on the level of relative abundance given that the microbe is present, respectively. That is, there is a conditional interpretation in Part II. However, it is often of great interest to have a straightforward interpretation of covariate effects on the overall marginal (unconditional) mean. For example, [13] proposed a marginalized two-part log-normal model by parameterizing covariates effects directly in terms of the marginal mean.

As conventional two-part Beta regression models do not provide an unconditional interpretation of covariate effects, we propose a marginalized two-part Beta regression model for microbiome abundance data which parameterizes covariate effects in terms of the marginal mean. The proposed model not only accounts for the zero-inflated nature of the microbiome data but also yields more interpretable effect estimates.

Of note, an alternative to describe zero-inflated data is the Tobit model [14] where zero values are considered as left censored observations of the underlying true negative values (of Normal or other distributions accommodating negative values). However, the Tobit model is not appropriate for the Beta distribution which does not have a support of negative values. Consequently, the Tobit model cannot be applied directly to the relative abundance data.

## Models

In the following Section, we will introduce the conventional two-part Beta regression model and the proposed marginalized two-part Beta regression model. We will also describe their properties to assess the overall impact of covariates on the marginal mean, and demonstrate that the proposed model outperforms the conventional model.

### Two-part Beta regression model

We begin with the conventional two-part model with a Beta component in Part II [10–12]. For a given operational taxonomic unit (OTU), let *Y*_*i*_ denote its semi-continuous relative abundance for subject *i*, where 0 ≤ *Y*_*i*_ < 1 and *i* = 1, 2, …, *n*. Specifically, a two-part Beta regression model has the following form:

$$\begin{array}{ccc} Y_{i} & ∼ & 0\;\;\text{with}\;\text{probability}\;\;1-p_{i}\\ & ∼ & \text{Beta}\left(\mu_{i}\phi ,\left(1-\mu_{i}\right)\phi \right)\;\;\text{with}\;\text{probability}\;\;p_{i}, \end{array}$$

where the density function of the Beta distribution is parameterized as

$$\frac{\Gamma (\phi )}{\Gamma \left(\mu_{i}\phi \right)\Gamma \left[\left(1-\mu_{i}\right)\phi \right]}y_{i}^{\mu_{i}\phi -1}{\left(1-y_{i}\right)}^{(1-\mu_{i})\phi -1},$$

with *μ*_*i*_ (0 < *μ*_*i*_ < 1) and *ϕ* (*ϕ* > 0) being the mean and dispersion parameters of the Beta distribution, respectively, and *p*_*i*_ is the probability that the observation *Y*_*i*_ is from the Beta distribution. The two-part model describes the probability *p*_*i*_ in the logistic component and the conditional mean in the Beta component as functions of covariates,

$$\text{logit}(p_{i})=\log\left(\frac{p_{i}}{1-p_{i}}\right)=X_{i}^{T}\alpha ,$$ (1)

$$\text{logit}\left(\mu_{i}\right)=\text{logit}\left[E\left(Y_{i}|Y_{i}>0\right)\right]=\log\left(\frac{\mu_{i}}{1-\mu_{i}}\right)=X_{i}^{T}\beta ,$$ (2)

where ***α*** and ***β*** are vectors of regression coefficients, ***X***_*i*_ = (1, *x*_*i*1_, …, *x*_*ip*_)^*T*^ is the (*p* + 1) dimensional covariate vector (including an intercept) for the *i*-th subject. We assume identical covariates for both parts of the model for simplicity of notation. One can instead allow for different sets of covariates for the two parts.

### Marginalized two-part Beta regression model

To obtain interpretable covariate effects on the marginal mean, we propose the following marginalized two-part Beta regression model. Let *v*_*i*_ = *E*(*Y*_*i*_) be the marginal mean of *Y*_*i*_. The first part of the proposed marginalized two-part model is the same as Part I in the conventional two-part model,

$$\begin{array}{c} \text{logit}\left(p_{i}\right)=\log\left(\frac{p_{i}}{1-p_{i}}\right)=X_{i}^{T}\alpha . \end{array}$$ (3)

In Part II, the marginal (unconditional) mean *v*_*i*_, instead of the conditional mean *μ*_*i*_, is modeled as a function of covariates:

$$\begin{array}{c} \text{logit}\left(v_{i}\right)=\log\left(\frac{v_{i}}{1-v_{i}}\right)=X_{i}^{T}\gamma . \end{array}$$ (4)

As we can see, the marginalized two-part model not only captures zero-inflation and skewness as the conventional two-part model, but also allows us to examine covariate effects on the overall marginal mean.

In the S1 Text, we can see that the likelihood of the conventional two-part model can be reparameterized to that of ***α***, ***γ*** and *ϕ* in the marginalized model. However, the interpretation of covariate effects are different in the two frameworks, which will be elaborated in the next subsection.

The estimation of the marginalized two-part model can be carried out in SAS Proc NLMIXED (The main code is shown in S1 Code). To obtain starting values of the estimation, a logistic model and a Beta regression model are fitted for the binary part and the positive part, respectively. Then the estimates of these two models are used as starting values for the two-part marginalized model. The convergence of the estimation is determined by a threshold value 1 × 10^−8^ for the relative gradient, a common convergence criterion in SAS Proc NLMIXED. This criterion is satisfied in our simulations for all replicates, and in the real data analysis for all 131 OTUs.

### Interpretation of covariate effects

#### For the conventional model

Using the conventional two-part model shown in Eqs (1) and (2), *β*_*j*_ is interpreted as the effect of a unit increase in the *j*th covariate on the logit of the conditional mean of *Y*_*i*_ given *Y*_*i*_ is positive. In many applications, however, the primary interest is to examine the impact of covariates on the overall marginal mean *E*(*Y*_*i*_). For the conventional two-part model, we have

$$\begin{array}{c} E\left(Y_{i}\right)=p_{i}E\left(Y_{i}|Y_{i}>0\right)=\frac{\exp(X_{i}^{T}\alpha )}{1+\exp(X_{i}^{T}\alpha )}\cdot \frac{\exp(X_{i}^{T}\beta )}{1+\exp(X_{i}^{T}\beta )}. \end{array}$$ (5)

Along the lines of [15], we can assess the effect of the *j*-th continuous covariate *x*_*ij*_ on the unconditional mean as

$$\begin{array}{c} \frac{\partial }{\partial x_{ij}}(\text{logit}[E\left(Y_{i}\right)])=\frac{\partial }{\partial x_{ij}}(\text{logit}[p\left(x_{ij}\right)\mu \left(x_{ij}\right)]), \end{array}$$ (6)

where

$$\begin{array}{ccc} p(x_{ij}) & = & \frac{\exp[x_{ij}\alpha_{j}+X_{i(-j)}^{T}\alpha_{\left(-j\right)}]}{1+\exp[x_{ij}\alpha_{j}+X_{i(-j)}^{T}\alpha_{\left(-j\right)}]},\\ \mu (x_{ij}) & = & \frac{\exp[x_{ij}\beta_{j}+X_{i(-j)}^{T}\beta_{\left(-j\right)}]}{1+\exp[x_{ij}\beta_{j}+X_{i(-j)}^{T}\beta_{\left(-j\right)}]}, \end{array}$$

with *α*_*j*_ and *β*_*j*_ being the coefficients corresponding to *x*_*ij*_ in the conventional two-part model and ***X***_*i*(−*j*)_, ***α***_(−*j*)_, and ***β***_(−*j*)_ be the corresponding vectors with the *j*-th covariate removed.

A straightforward calculation shows that (6) can be equivalently written as

$$\begin{array}{c} \frac{\partial }{\partial x_{ij}}(\text{logit}[E\left(Y_{i}\right)])=c_{1}\left(\alpha_{j},\beta_{j}\right)\alpha_{j}+c_{2}\left(\alpha_{j},\beta_{j}\right)\beta_{j}, \end{array}$$ (7)

where

$$\begin{array}{ccc} c_{1}\left(\alpha_{j},\beta_{j}\right) & = & \frac{1-p(x_{ij})}{1-p\left(x_{ij}\right)\mu \left(x_{ij}\right)},\\ c_{2}\left(\alpha_{j},\beta_{j}\right) & = & \frac{1-\mu (x_{ij})}{1-p\left(x_{ij}\right)\mu \left(x_{ij}\right)}. \end{array}$$

As the logit transformation is a monotonically increasing function in the interval (0, 1), the hypothesis test of the covariate effects on the marginal mean is equivalent to that on its logit transformation. In Eq (7), the logit transformation of the marginal mean abundance is independent of covariate *x*_*ij*_ if both *α*_*j*_ and *β*_*j*_ are zero. However, if *α*_*j*_ and *β*_*j*_ have opposite signs, even when they are not zero, the logit transformation of the marginal mean abundance may be still independent of covariate *x*_*ij*_. Furthermore, the coefficients *c*_1_(*α*_*j*_, *β*_*j*_) and *c*_2_(*α*_*j*_, *β*_*j*_) in Eq (7) are functions of *α*_*j*_ and *β*_*j*_. Thus, the independence between the marginal mean and covariate *x*_*ij*_ cannot be tested simply as the hypothesis of *α*_*j*_ = 0 and *β*_*j*_ = 0, e.g., by the likelihood ratio test. Instead, the Delta method has to be used on the hypothesis test of Eq (7), which depends on ***X***_*i*(−*j*)_ in a complicated way.

When the interest is to assess the effect of a discrete variable on response, e.g., placebo vs. treatment, Eq (7) no longer applies. Without loss of generality, consider a binary covariate *x*_*ik*_ taking value 0 or 1. Similar to [15], the difference in the logit transformation of the marginal mean with *x*_*ik*_ = 1 vs. *x*_*ik*_ = 0 is used to evaluate the impact on the expected marginal mean response.

Under the conventional two-part model, the difference between the logit transformations with *x*_*ik*_ = 1 and *x*_*ik*_ = 0 is

$$\begin{array}{ccc} & & \text{logit}\left[E\left(Y_{i}|x_{ik}=1\right)\right]-\text{logit}\left[E\left(Y_{i}|x_{ik}=0\right)\right]\\ & = & \text{logit}\left[p\left(x_{ik}=1\right)\mu \left(x_{ik}=1\right)\right]-\text{logit}\left[p\left(x_{ik}=0\right)\mu \left(x_{ik}=0\right)\right]\\ & = & \alpha_{k}+\beta_{k}+b_{1}\left(\alpha_{k}\right)+b_{2}\left(\beta_{k}\right)+b_{3}\left(\alpha_{k},\beta_{k}\right), \end{array}$$ (8)

where

$$\begin{array}{ccc} b_{1}\left(\alpha_{k}\right) & = & \ln(\frac{1+\exp[X_{i(-k)}^{T}\alpha_{\left(-k\right)}]}{1+\exp[\alpha_{k}+X_{i(-k)}^{T}\alpha_{\left(-k\right)}]}),\\ b_{2}\left(\beta_{k}\right) & = & \ln(\frac{1+\exp[X_{i(-k)}^{T}\beta_{\left(-k\right)}]}{1+\exp[\beta_{k}+X_{i(-k)}^{T}\beta_{\left(-k\right)}]}),\\ b_{3}\left(\alpha_{k},\beta_{k}\right) & = & \ln(\frac{1-p\left(x_{ik}=0\right)\mu \left(x_{ik}=0\right)}{1-p\left(x_{ik}=1\right)\mu \left(x_{ik}=1\right)}). \end{array}$$

It is worth noting that *b*_1_(*α*_*k*_), *b*_2_(*β*_*k*_), and *b*_3_(*α*_*k*_, *β*_*k*_) all equal to 0 if *α*_*k*_ and *β*_*k*_ are 0. Similar to the continuous covariate, the logit transformation of the marginal mean abundance does not depend on the binary covariate *x*_*ik*_ if both *α*_*k*_ and *β*_*k*_ are zero. However, even though neither of the coefficients is zero, the transformed mean abundance may still be independent of the binary covariate *x*_*ik*_ when *α*_*k*_ and *β*_*k*_ have opposite signs. Eq (8) indicates that the independence between the response and the binary covariate *x*_*ik*_ cannot be ascertained by directly testing *α*_*k*_ = 0 and *β*_*k*_ = 0 by e.g., the likelihood ratio test, as shown in the simulation studies and the real data analysis.

#### For the marginalized model

In the marginalized two-part model Eqs (3) and (4), the effect of a continuous covariate *x*_*ij*_ on the marginal mean *E*(*Y*_*i*_) can be characterized by

$$\begin{array}{c} \frac{\partial }{\partial x_{ij}}(\text{logit}[E\left(Y_{i}\right)])=\gamma_{j}, \end{array}$$ (9)

where *γ*_*j*_ is the coefficient corresponding to *x*_*ij*_ in Eq (4). Thus, the effect of the covariate *x*_*ij*_ on the marginal mean abundance is determined by its coefficient in the marginalized model. With the marginalized two-part model, we can estimate the coefficient *γ*_*j*_ as well as test the effect on the marginal mean.

As for a binary covariate in the marginalized two-part model, the difference in logit transformation of the marginal mean with *x*_*ik*_ = 1 vs. *x*_*ik*_ = 0 can be expressed as

$$\begin{array}{c} \text{logit}\left[E\left(Y_{i}|x_{ik}=1\right)\right]-\text{logit}\left[E\left(Y_{i}|x_{ik}=0\right)\right]=\gamma_{k} \end{array}$$ (10)

One can see that the effect of a binary covariate *x*_*ik*_ on the marginal mean abundance is determined by its coefficient *γ*_*k*_ in the marginalized two-part model. The logit transformation of the marginal mean abundance with *x*_*ik*_ = 1 is bigger than that with *x*_*ik*_ = 0 when *γ*_*k*_ is positive, and the reverse is true when *γ*_*k*_ is negative.

## Results

In this section, simulation studies and real data analysis are presented to assess the performance of the proposed marginalized and the conventional two-part models. Results show that the proposed model outperforms the conventional model, which is consistent with the theoretical results.

### Simulation studies

In this section, we conduct simulation studies to evaluate the finite-sample performance of the proposed marginalized two-part model. To test the effect of the covariate on the overall marginal mean *E*(*Y*_*i*_), likelihood ratio tests (LRT) are performed and compared under the marginalized two-part (MTP) model and the conventional two-part (CTP) model. In addition, the two sample T-test and the Wilcoxon rank sum test are also compared.

We assume that, in both parts, there is only one binary covariate *x*_1_, which is generated from the Bernoulli distribution with *p* = 0.5. However, according to the interpretation of the covariate effects in the preceding section, the proposed model can be applied to multiple covariates. The response *y*_*i*_ is generated below:

$$\begin{array}{cc} \text{logit}\left(p_{i}\right) & =\alpha_{0}+\alpha_{1}x_{i1},\\ \text{logit}\left(v_{i}\right) & =\gamma_{0}+\gamma_{1}x_{i1},\\ f\left(y_{i}\right) & ={\left(1-p_{i}\right)}^{1_{\left(y_{i}=0\right)}}\times {\left[p_{i}\text{Beta}\left(\mu_{i}\phi ,\left(1-\mu_{i}\right)\phi \right)\right]}^{1_{\left(y_{i}>0\right)}}, \end{array}$$

where $\mu_{i}=\frac{1+\exp(-x_{i}^{T}\alpha )}{1+\exp(-x_{i}^{T}\gamma )}$ is the conditional mean given that *y*_*i*_ is positive and *ϕ* is the dispersion parameter of the Beta distribution.

In the simulation studies, 1000 samples of sizes 200 and 400 are generated. We set the parameters as *α*_0_ = 1.5, *γ*_0_ = −2.5, and *ϕ* = 1, while *α*_1_ and *γ*_1_ may have different values according to which of the two criteria are under study: the type I error or the power.

First, we evaluate the type I error for testing the null hypothesis *H*_0_: *the binary covariate x_1_ has no effect on the overall marginal mean of y_i_*. In the MTP model, this is equivalent to testing $H_{0}^{M}:\gamma_{1}=0$ as shown in Eq (10). However, testing $H_{0}^{C}:\alpha_{1}=\beta_{1}=0$ in the CTP model is not equivalent to testing *H*_0_. Specifically, according to Eq (8), even though neither of the coefficients is zero, the binary covariate *x*_1_ may still have no effect on the marginal mean. This means that the conventional model cannot control the type I error for testing *H*_0_ when both *α*_1_ and *β*_1_ are non-zero.

**Fig 1:**
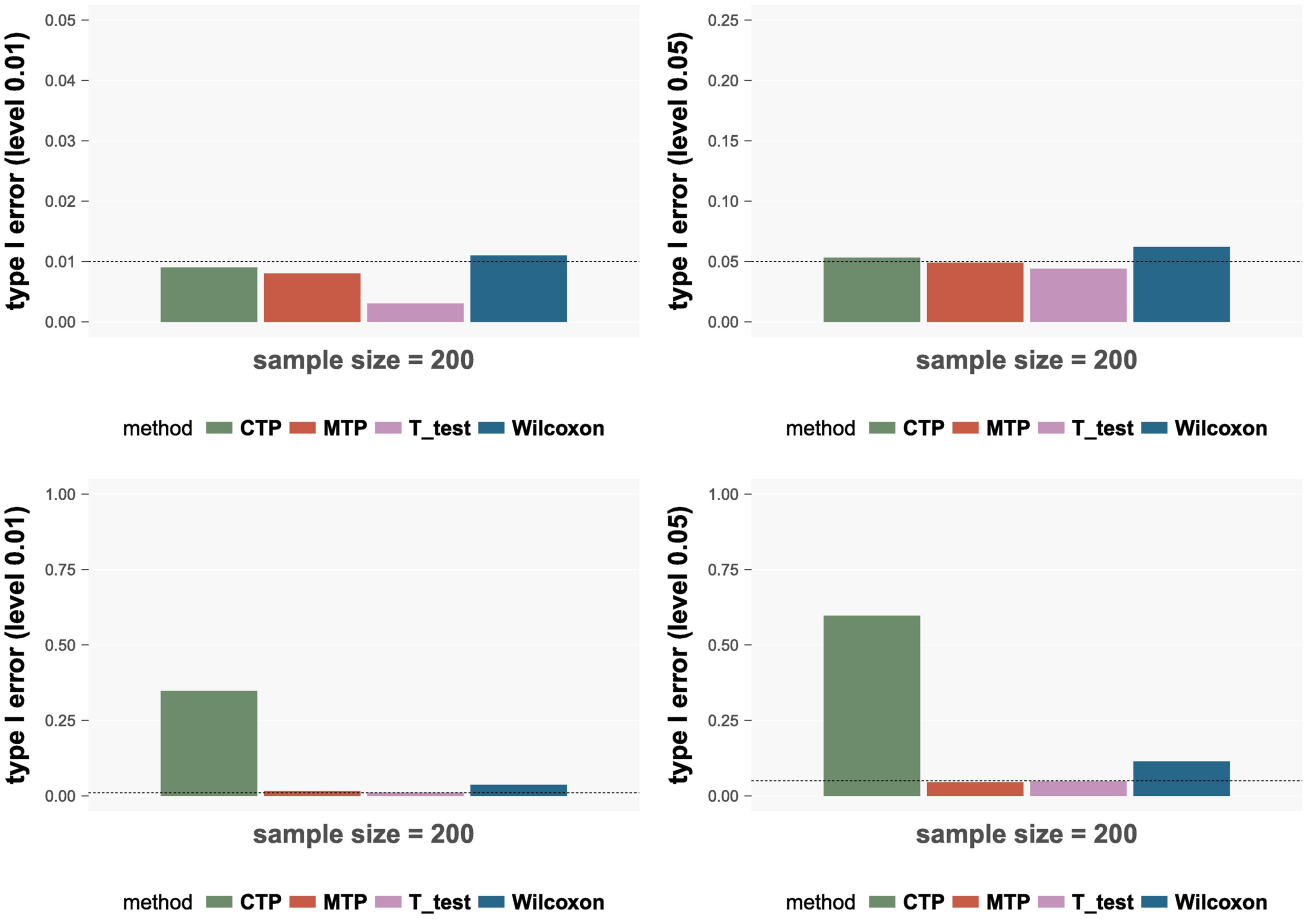
Type I errors of the four methods. The results in the upper panels correspond to the setting *α*_1_ = 0, *γ*_1_ = 0 and the lower panels correspond to setting *α*_1_ = 1, *γ*_1_ = 0. In each setting, the left panel shows the results for significance level 0.01 and the right panel shows the results for level 0.05. The dashed horizontal line in each panel represents the correct level. The results for sample size 400 can be found in S1 Fig in Supporting information.

The results are shown in Fig 1. Type I errors are calculated under two settings: *α*_1_ = 0, *γ*_1_ = 0 and *α*_1_ = 1, *γ*_1_ = 0. For each setting, two *α*-levels are considered: 0.01 and 0.05. As we can see from Fig 1, under the first setting (*α*_1_ = 0, *γ*_1_ = 0), all the methods control the type I error reasonably well. Under the setting *α*_1_ = 1, *γ*_1_ = 0, the LRT under the MTP and the T-test control the type I error well, while the LRT under the CTP and the Wilcoxon test cannot control the type I error, especially the LRT under the CTP model. Because in this setting, testing $H_{0}^{C}$ in the CTP model is not equivalent to testing the null hypothesis *H*_0_.

**Fig 2:**
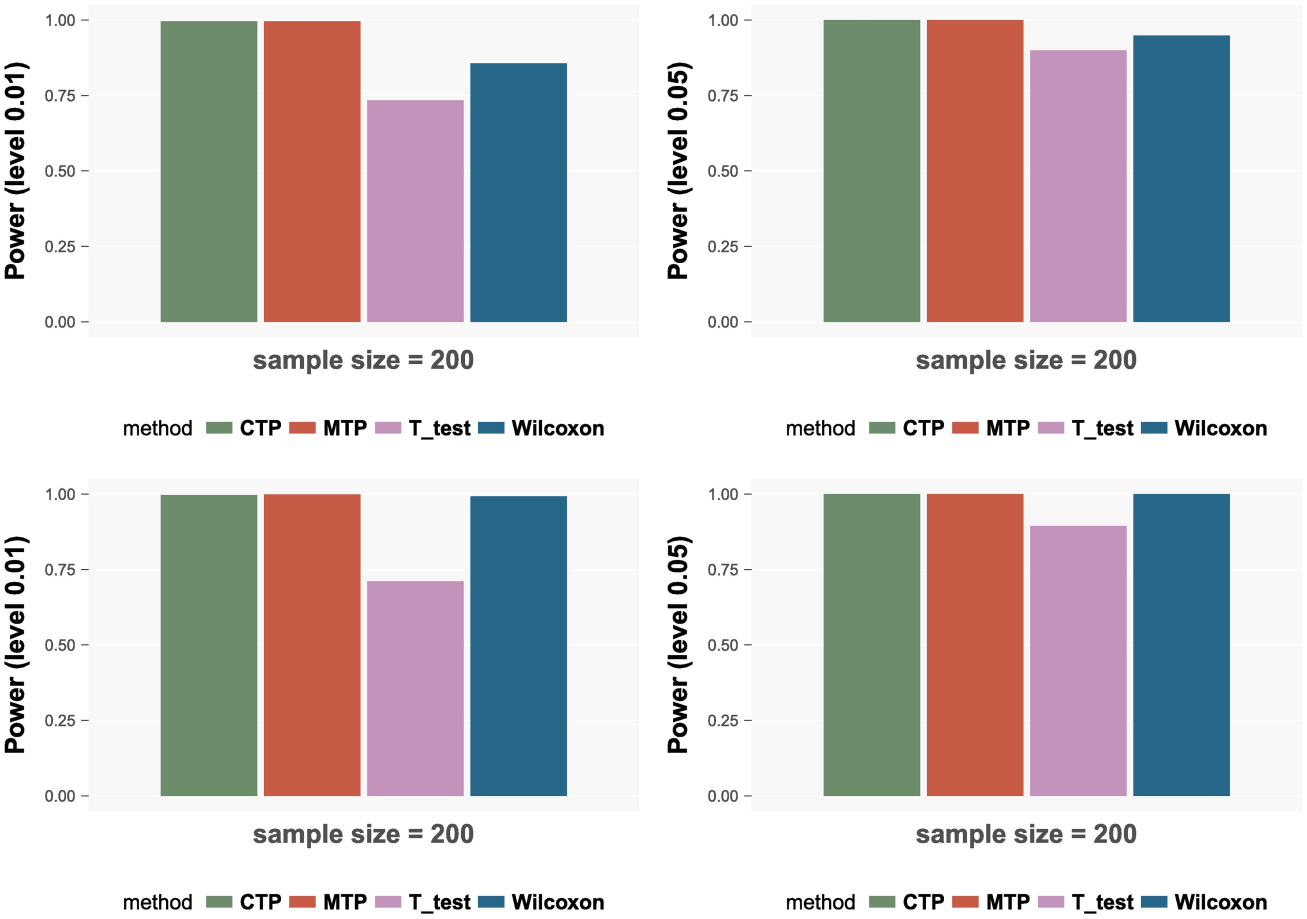
Powers of the four methods. The upper panels show the powers corresponding to the setting *α*_1_ = 0, *γ*_1_ = 1 and the lower panels show the powers corresponding to the setting *α*_1_ = 1, *γ*_1_ = 1. In each setting, the left panel shows the results for significance level 0.01 and the right panel shows the results for level 0.05. The powers for sample size 400 are shown in S2 Fig in Supporting information.

The powers under two different settings, *α*_1_ = 0, *γ*_1_ = 1 and *α*_1_ = 1, *γ*_1_ = 1, are shown in Fig 2. As we can see, the LRT under the CTP and the MTP are the most powerful methods with the power close to 1 in all settings. The Wilcoxon test performs a little worse than the LRT while the T-test has the lowest power.

We also estimate the coefficients in the MTP model under the setting *α*_1_ = 1, *γ*_1_ = 1. The results in Table 1 demonstrate that the biases are negligible and the coverage probabilities are acceptably close to the nominal level 0.95 for all the model parameters. In addition, we observe small differences between the empirical standard errors and our estimates. The mean squared errors for sample size 400 are smaller than those for sample size 200.

**Table 1:** Estimates of the coefficients in the marginalized two-part model under the setting *α*_1_ = 1, *γ*_1_ = 1.

Parameter	sample size = 200					sample size = 400				
	Est	SE	SEM	CP	MSE	Est	SE	SEM	CP	MSE
*α*_0_ = 1.5	1.5321	0.2695	0.2646	0.955	0.0736	1.5153	0.1921	0.1854	0.943	0.0375
*α*_1_ = 1	1.0345	0.4876	0.4803	0.960	0.2387	1.0078	0.3457	0.3320	0.947	0.1195
*γ*_0_ = -2.5	-2.5104	0.1673	0.1727	0.956	0.0281	-2.5074	0.1210	0.1219	0.949	0.0147
*γ*_1_ = 1	0.9962	0.1758	0.1803	0.949	0.0309	1.0002	0.1253	0.1273	0.957	0.0157
*ϕ* = 1	1.0323	0.1374	0.1331	0.956	0.0199	1.0157	0.0929	0.0923	0.954	0.0089

Est: mean of the parameter estimates;

SE: standard error of the parameter estimates;

SEM: sample mean of the standard error estimates;

CP: coverage probability of the corresponding 95% confidence interval.

According to the simulation results, the LRT under the MTP model has the best performance: it controls the type I error reasonably well and also achieves the best power. The T-test has the similar performance in the error control while it is not as powerful as the LRT under the MTP model. The LRT under the CTP model is powerful, however, it fails to control the type I error. The Wilcoxon test has poor performances in both the error control and power than the LRT under the MTP model.

To assess the robustness of the proposed method, we consider a setting where positive responses are generated from another distribution. First of all, the only covariate *x*_*i*_ is generated from the Uniform distribution on (0, 1), while the response *y*_*i*_ has the following distribution:

$$\begin{array}{c} y_{i}\;∼\;0\;\;\text{with}\;\text{probability}\;\;1-p_{i}, \end{array}$$

where

$$\begin{array}{c} \text{logit}\left(p_{i}\right)=\alpha_{0}+\alpha_{1}x_{i}; \end{array}$$

and the overall marginal mean *v*_*i*_ of the response is

$$\begin{array}{c} \text{logit}\left(v_{i}\right)=\gamma_{0}+\gamma_{1}x_{i}. \end{array}$$

Instead of the Beta distribution, positive responses are generated from the Binomial distribution Bin(100, *μ*_*i*_) and then divided by 100 to make them bounded in (0, 1). As in the previous simulation, we set $\mu_{i}=\frac{1+\exp(-x_{i}^{T}\alpha )}{1+\exp(-x_{i}^{T}\gamma )}$. The probability of having exactly 0 success in 100 trials is (1 − *μ*_*i*_)^100^, which is negligible with the proper choice of the parameters ***α*** and ***γ***. Thus almost all the zero values in this zero-inflated Binomial data are structural zeros.

In this simulation study, 1000 samples of sizes 200 and 400 are generated. The parameters are set as *α*_0_ = 2, *γ*_0_ = −0.5, while *α*_1_ and *γ*_1_ may have different values in order to calculate the type I errors and the powers.

**Fig 3:**
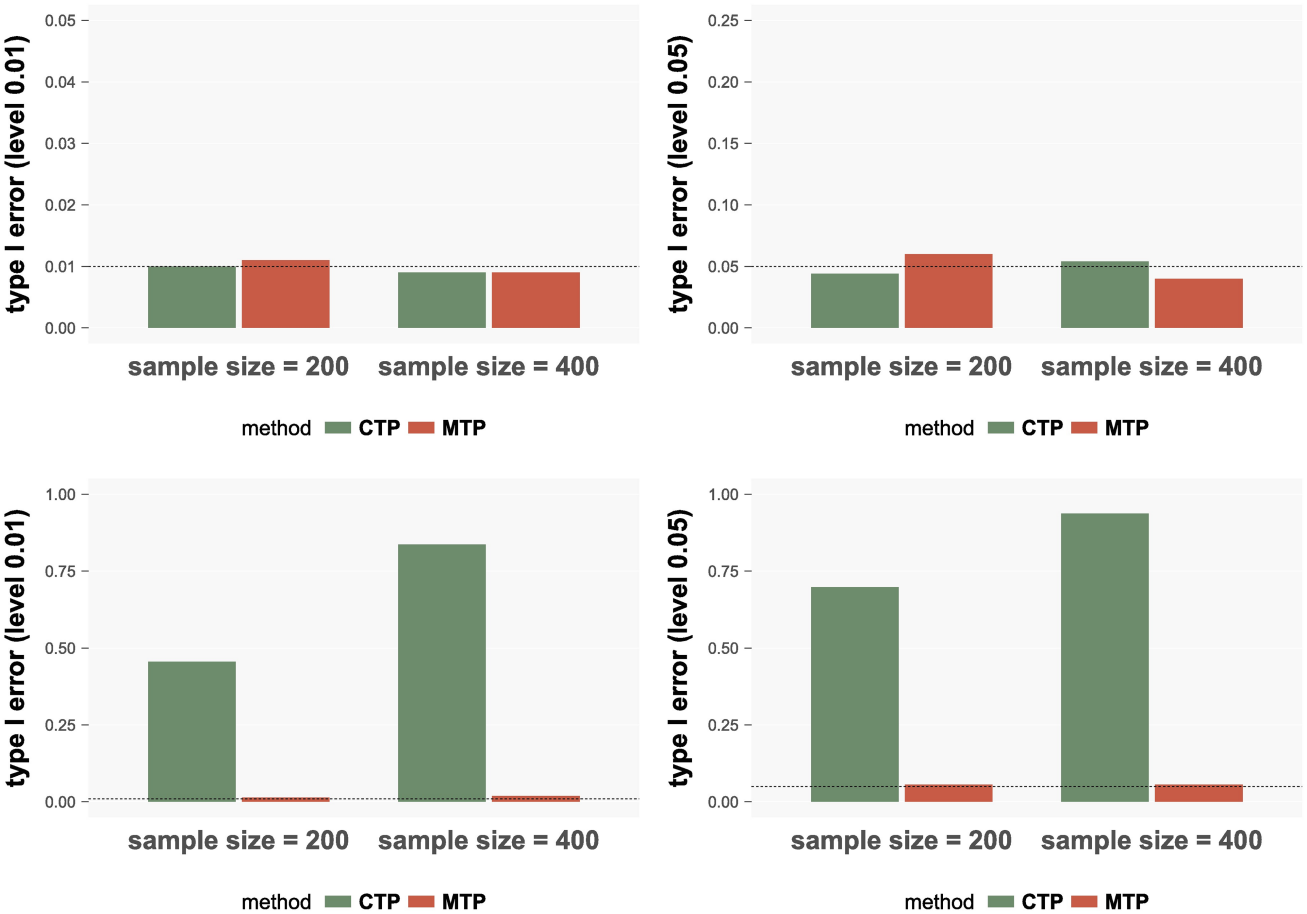
Type I errors for the CTP model and the MTP model. The results in the upper panels correspond to the setting *α*_1_ = 0, *γ*_1_ = 0 and the lower panels correspond to the setting *α*_1_ = 1, *γ*_1_ = 0. In each setting, the left panel shows the results for significance level 0.01 and the right panel shows the results for level 0.05. The dashed horizontal line in each panel represents the correct *α*-level.

The type I errors are calculated under two settings: *α*_1_ = 0, *γ*_1_ = 0 and *α*_1_ = 1, *γ*_1_ = 0. For each setting, two *α*-levels are considered: 0.01 and 0.05. As we can see from Fig 3, under both settings, the proposed marginalized model controls the type I error reasonably well. The conventional model controls the type I error under the setting *α*_1_ = 0, *γ*_1_ = 0 while it fails under the setting *α*_1_ = 1, *γ*_1_ = 0, similar to Fig 1.

**Fig 4:**
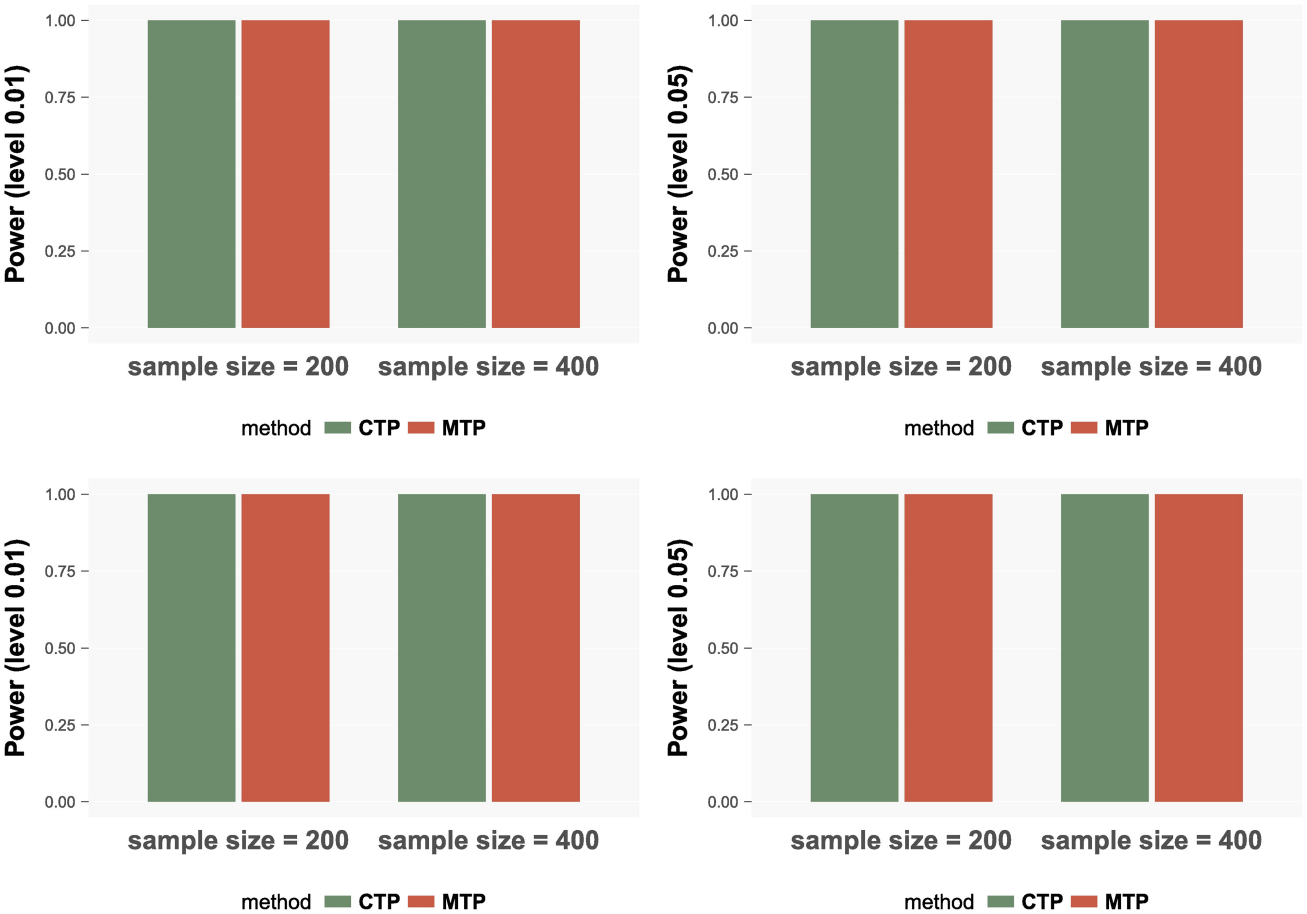
Powers for the CTP model and the MTP model. The powers in the upper panels correspond to the setting *α*_1_ = 0, *γ*_1_ = 1 and the lower panels correspond to setting *α*_1_ = 1, *γ*_1_ = 1. In each setting, the left panel shows the results for significance level 0.01 and the right panel shows the results for level 0.05.

As shown in Fig 4, both the marginalized model and the conventional model have power equal to 1 under all settings.

From the simulation studies we can conclude that the proposed marginalized two-part Beta regression model is powerful and control the type I error well. Also, it is robust against model misspecification.

### Real data analysis

In this section, the proposed marginalized two-part model and the conventional two-part model are applied to a real metagenomic dataset on mouse skin microbiota to investigate the effects of immunization on the relative abundances of 131 core OTUs [16, 17]. The data are publicly available at https://www.nature.com/articles/ncomms3462#supplementary-information. In addition to the likelihood ratio tests under CTP and MTP, the T test and the Wilcoxon rank sum test are also included for comparison. All the tests are carried out with Bonferroni’s correction.

The skin dataset contains the relative abundances of the most common 131 OTUs for 261 mouse skin samples, including 78 non-immunized and 183 immunized individuals. There is a presence of a large portion of zero abundances in the skin data, ranging from 0 to 68.97% with average 33.03% and median 33.72% (see S3 and S4 Figs). The positive values are highly right skewed and the logit transformations in the MTP model and the CTP model capture the skewness (See S5 Fig).

**Fig 5:**
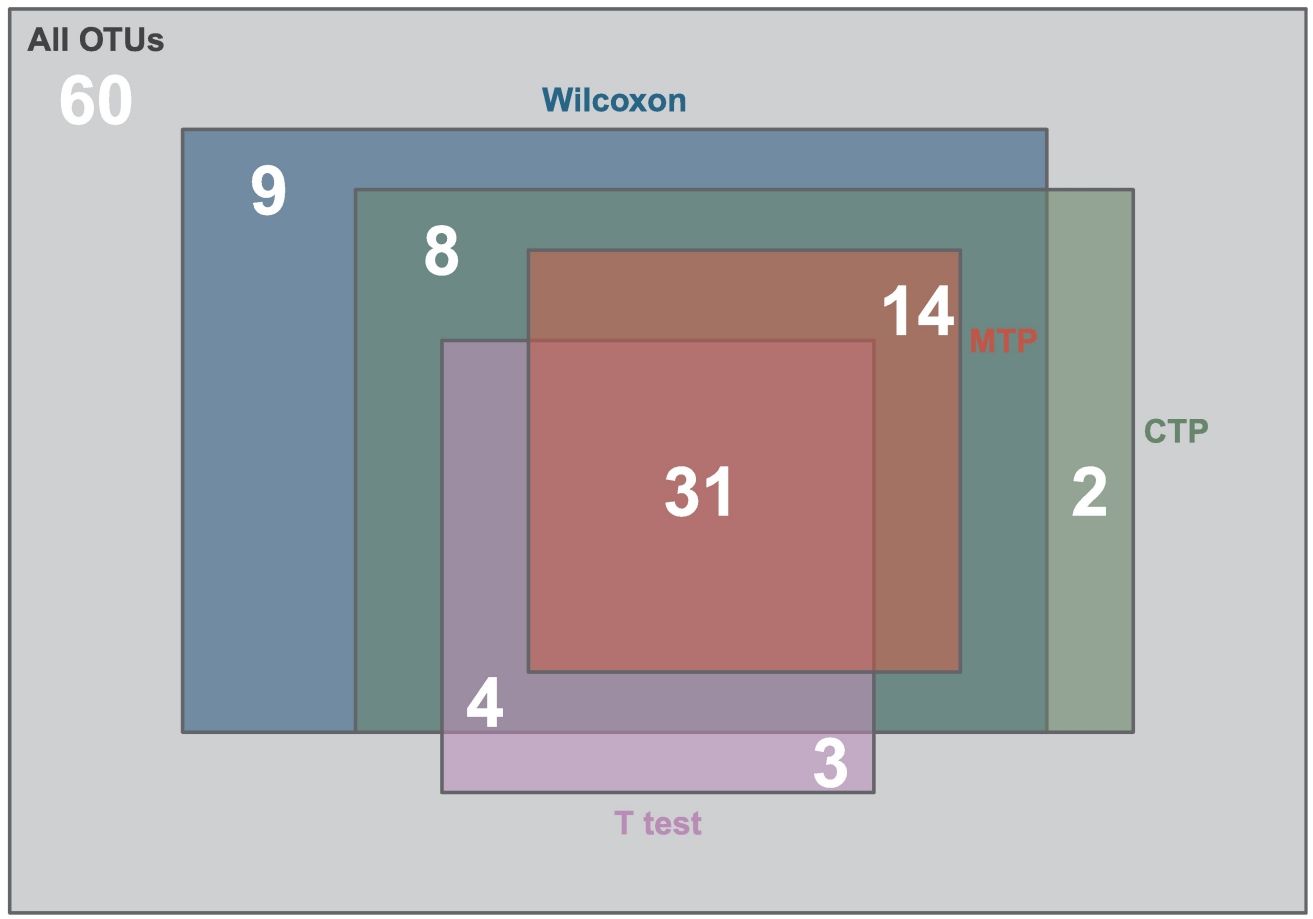
Venn diagram for the OTUs. Among all the 131 OTUs, 60 OTUs are not identified by any methods and the other 71 OTUs are identified by at least one method. For example, “31” in the intersection of all sets indicates that 31 OTUs are identified by all methods; while “4” located in the intersection of three sets, indicates that 4 OTUs are identified by three methods, namely, the T test, the CTP model, and the Wilcoxon test.

Fig 5 shows the results for these four methods. As we can see, the LRT under the marginalized two-part model results in significant effects of immunization on 45 (namely, 31 + 14) OTUs. The LRT under the conventional two-part model has significant results for all these 45 OTUs, and 14 (namely, 8 + 4 + 2) additional OTUs. The T test identifies 31 of these 45 OTUs and another 7 (namely, 4 + 3) OTUs. Similar to the LRT under conventional two-part model, the Wilcoxon test identifies all these 45 OTUs and 21 (namely, 9 + 8 + 4) additional OTUs. Finally, 60 OTUs are not identified by any methods.

The LRT under the CTP model and the Wilcoxon test identify more OTUs than the LRT under the MTP due to their failure to control the type I error as shown in Simulation studies (Fig 1). Actually, for those 14 OTUs identified by the CTP but not by the MTP, all of them have significant coefficients in Part I of the two-part model. Out of the 21 OTUs that are identified by the Wilcoxon test but not by the MTP, 17 have significant coefficients in Part I of the two-part model. This corresponds to the setting *α*_1_ = 1, *γ*_1_ = 0 where both the CTP and the Wilcoxon test have much higher type I errors than the MTP (See the lower panel of Fig 1). Because it is less powerful than the MTP (Fig 2), the T test identifies less OTUs than the MTP.

Table 2 shows 10 most significant OTUs from the MTP model. As in [17], for OTUs which cannot be classified at the species level, the next highest classifiable taxonomic level (denoted by ‘o’, ‘f’ and ‘g’ for order, family, and genus, respectively) is displayed. We use a number in the superscript to distinguish among different OTUs with the same classification name. The detailed results of all the 45 OTUs identified by the proposed MTP model are shown in S1 Table.

**Table 2:** Top 10 OTUs identified by the MTP model.

Rank	ID	Species	Est	SE	p Value
1	237040	g_Alicyclobacillus	2.3262	0.3148	< 1E-16
2	101810	g_Helicobacter^1^	-1.3934	0.1460	< 1E-16
3	52884	g_Helicobacter^2^	1.9091	0.2875	4.88E-15
4	N10167	o_Bacteroidales^1^	2.5761	0.4234	8.33E-15
5	381715	f_Ruminococcaceae	2.7674	0.4777	6.82E-14
6	269548	g_Helicobacter^3^	1.2357	0.1833	3.57E-13
7	N26397	Acetobacter aceti	1.8841	0.3055	4.86E-13
8	294146	Acetobacterorleanensis	2.0662	0.3387	5.66E-13
9	N2007	Acinetobacterlwoffii	2.5549	0.4498	1.19E-12
10	N8891	g_Mucispirillum	1.5868	0.2679	1.53E-11

Est: estimation of the coefficient of treatment in the second submodel;

SE: standard error of the coefficient of treatment in the second submodel.

**Fig 6:**
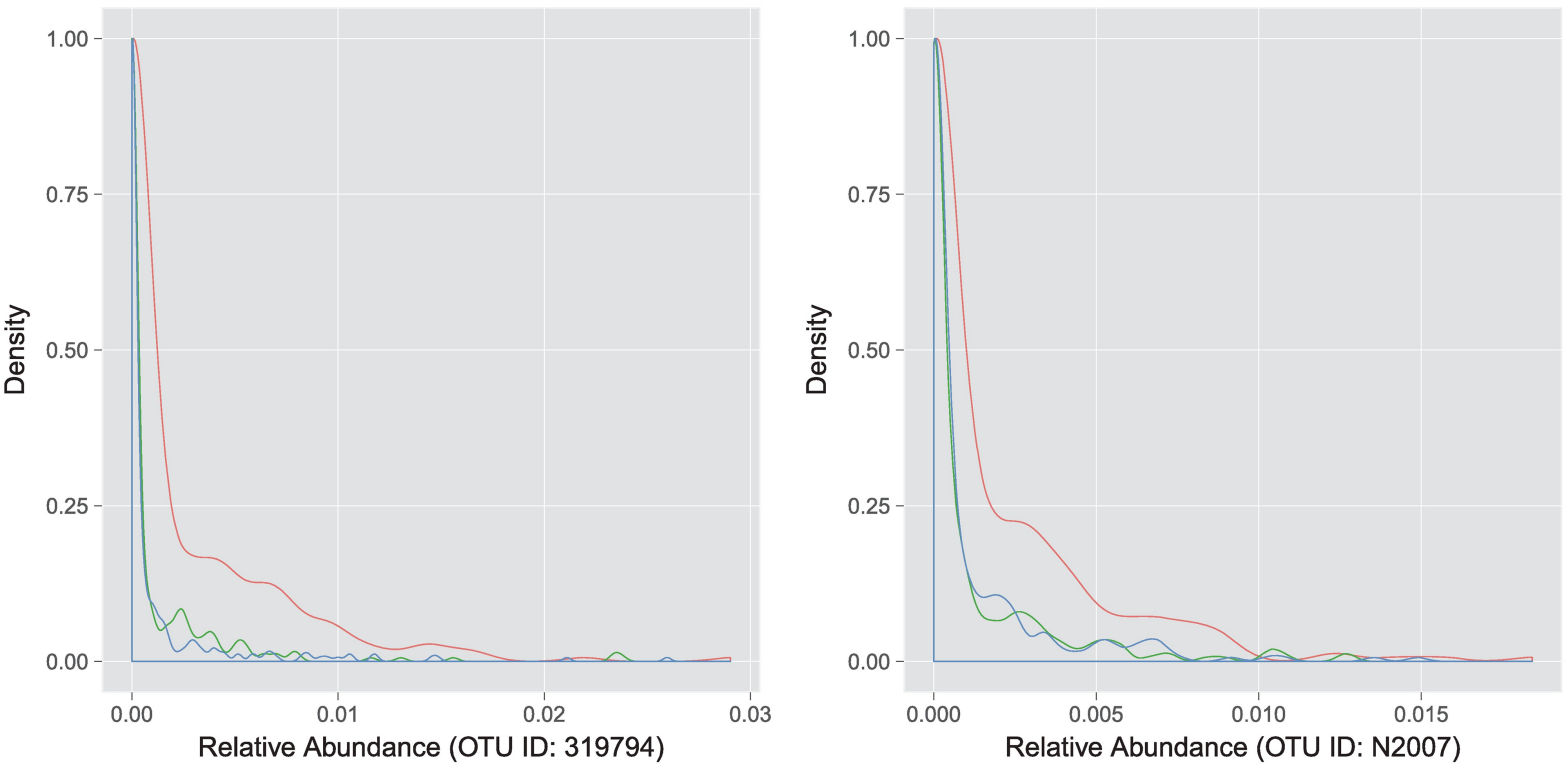
Density curves for two OTUs. The blue curve shows the density of the observed data. The green curve shows the density of predictions from the MTP model while the red curve represents the density of predictions from the CTP model.

Moreover, for most of the 131 OTUs, the proposed marginalized two-part model fits the observed data better than the conventional two-part model. Fig 6 shows the density curves of the observed relative abundances, the predicted relative abundances using the MTP model, and the predicted relative abundances using the CTP model for two OTUs. As we can see, the MTP model fits the observed data much better than the CTP model.

## Discussion

In this paper, we propose a marginalized two-part Beta regression model for semi-continuous microbiome compositional data. This model allows investigators to obtain covariate effects on the marginal mean of the outcome. It takes into account the compositional and zero-inflation nature of the microbiome relative abundance data. It also has an unconditional interpretation of the covariate effect on the marginal mean. Our proposed marginalized two-part model has satisfactory performance in both simulation studies and real data analysis.

For count outcomes exhibiting many zeros, a zero-inflated Poisson (ZIP) regression model or a zero-inflated negative binomial (ZINB) model, is often employed to examine the relation between covariates and the response. To model the overall population mean count directly, the marginalized ZIP model and the marginalized ZINB model were proposed by [18] and [19], respectively. However, in the case of bounded count data, the ZIP is questionable while the zero-inflated binomial (ZIB) model and its extension for over-dispersion: the zero-inflated beta-binomial (ZIBB) model, are available in [20–22]. It is of interest to develop a marginalized modeling approach for ZIB or ZIBB.

More recently, there has been increasing interest in analyzing correlated zero-inflated semi-continuous data. The correlation may stem from the structure of clustered data or from longitudinal data where repeated measures are correlated for the same subject. Typically, random effects are included to account for the correlations between observations [10, 15, 23–25]. However, similar limitation exists in these two-part random effects models, as they cannot account for covariate effects on the marginal mean. Recently, Smith et al. [26] proposed a marginalized two-part model for longitudinal semicontinuous data based on the log-skew normal distribution for positive values. In future studies, we will extend our marginalized two-part model to correlated semi-continuous data bounded by 0 and 1.

Finally, it is of interest to consider different microbiomes together, taking into account the constraint that the relative abundances of all OTUs sum to 1. Scealy and Welsh [27, 28] considered Kent models for such compositional data. It merits further consideration to incorporate zero values in the Kent model framework.

## Supporting information

S1 Text Likelihood derivation. (PDF)

S1 Code SAS code. The main SAS codes for the conventional two-part model and the proposed marginalized two-part model are shown in this section.
(PDF)

S1 Fig Type I errors for the sample size 400. This figure shows the type I errors of the four methods for sample size 400. The results in the upper panels correspond to the setting *α*_1_ = 0, *γ*_1_ = 0 and the lower panels correspond to setting *α*_1_ = 1, *γ*_1_ = 0. In each setting, the left panel shows the results for significance level 0.01 and the right panel shows the results for significance level 0.05. The dashed horizontal line in each panel represents the significance level.
(TIF)

S2 Fig Powers for the sample size 400. This figure shows the powers of the four methods for sample size 400. The upper panel contains the power corresponding to the setting *α*_1_ = 0, *γ*_1_ = 1 and the lower panel shows the power corresponding to the setting *α*_1_ = 1, *γ*_1_ = 1. In each setting, the left figure shows the results for significance level and the right panel shows the results for significance level 0.05.
(TIF)

S3 Fig Zero-inflation of the skin data. The figure shows the distributions of relative abundances of 6 OTUs. From the upper panel to the lower panel and from the left to the right, the proportions of zero values for these 6 OTUs are 0.77%, 3.45%, 4.97%, 14.18%, 29.89%, and 48.28%, respectively.
(TIF)

S4 Fig The figure shows the percentages of zero abundance in the 261 mouse skin samples for all 131 core OTUs. The lower quartile and the upper quartile of the percentages are 20.11% and 48.28%, respectively.
(TIF)

S5 Fig Skewness of the skin data. The figure shows the histogram of the relative abundance for 6 OTUs. The first one in every panel is the histogram of the OTU in the original scale, while the second one in every panel shows the histogram after logit transformation.
(TIF)

S1 Table The detailed results of the MTP model. The table shows the detailed results of all the 45 OTUs that are identified by the proposed MTP model.
(PDF)
