# Supplementary material for: A marginalized two-part Beta regression model for microbiome compositional data
Source: PLoS Comput Biol. 2018 Jul 23;14(7):e1006329. doi: 10.1371/journal.pcbi.1006329 (PMC6072097; doi:10.1371/journal.pcbi.1006329)
Supplement: S1 Text — (PDF) [file pcbi.1006329.s001.pdf]

S1 Text: Likelihood Derivation.

In this Appendix, we give the log-likelihood for the proposed marginalized two-part model. First, when parameterized in terms of p_i, μ_i and ϕ, the log-likelihood is

$$\begin{aligned} l(\boldsymbol{p}, \boldsymbol{\mu}, \phi|\boldsymbol{y}) &= \sum_{i:y_i>0} \left\{ \ln(p_i) + g(\mu_i, \phi) + (\mu_i\phi - 1)\ln(y_i) + [(1-\mu_i)\phi - 1]\ln(1-y_i) \right\} \\ &+ \sum_{i:y_i=0} \ln(1-p_i), \end{aligned} \tag{S1}$$

where

$$g(\mu_i, \phi) = \ln\Gamma(\phi) - \ln\Gamma(\mu_i\phi) - \ln\Gamma((1-\mu_i)\phi). \tag{S2}$$

Notice the relationship between $v_i = E(Y_i)$ and $\mu_i = E(Y_i|Y_i > 0)$,

$$v_i = E(Y_i) = p_i E(Y_i|Y_i > 0) = p_i\mu_i. \tag{S3}$$

This gives rise to

$$\mu_i = \frac{v_i}{p_i} = \frac{1 + \exp(-\boldsymbol{X}_i^T\boldsymbol{\alpha})}{1 + \exp(-\boldsymbol{X}_i^T\boldsymbol{\gamma})}. \tag{S4}$$

Note also that

$$\begin{aligned} \ln(p_i) &= \boldsymbol{X}_i^T\boldsymbol{\alpha} - \ln[1 + \exp(\boldsymbol{X}_i^T\boldsymbol{\alpha})], \\ \ln(1-p_i) &= -\ln[1 + \exp(\boldsymbol{X}_i^T\boldsymbol{\alpha})]. \end{aligned} \tag{S5}$$

Thus we can express the log-likelihood in terms of $\boldsymbol{\alpha}$, $\boldsymbol{\gamma}$ and ϕ :

$$\begin{aligned} l(\boldsymbol{\alpha}, \boldsymbol{\gamma}, \phi|\boldsymbol{y}) &= \sum_{i:y_i=0} -\ln(1 + \exp(\boldsymbol{X}_i^T\boldsymbol{\alpha})) \\ &+ \sum_{i:y_i>0} \Bigg\{ \boldsymbol{X}_i^T\boldsymbol{\alpha} - \ln[1 + \exp(\boldsymbol{X}_i^T\boldsymbol{\alpha})] + h(\boldsymbol{\alpha}, \boldsymbol{\gamma}, \phi) \\ &+ \frac{1 + \exp(-\boldsymbol{X}_i^T\boldsymbol{\alpha})}{1 + \exp(-\boldsymbol{X}_i^T\boldsymbol{\gamma})}\phi\text{logit}(y_i) + \phi\ln(1-y_i) - \ln[y_i(1-y_i)] \Bigg\} \qquad \text{(S6)} \\ &= -\sum_i \ln(1 + \exp(\boldsymbol{X}_i^T\boldsymbol{\alpha})) \\ &+ \sum_{y_i>0} \Bigg\{ \boldsymbol{X}_i^T\boldsymbol{\alpha} + h(\boldsymbol{\alpha}, \boldsymbol{\gamma}, \phi) + \frac{1 + \exp(-\boldsymbol{X}_i^T\boldsymbol{\alpha})}{1 + \exp(-\boldsymbol{X}_i^T\boldsymbol{\gamma})}\phi\text{logit}(y_i) \\ &+ \phi\ln(1-y_i) - \ln[y_i(1-y_i)] \Bigg\}, \qquad \text{(S7)} \end{aligned}$$

where

$$\begin{aligned} h(\boldsymbol{\alpha}, \boldsymbol{\gamma}, \phi) &\doteq g\{\mu_i(\boldsymbol{\alpha}, \boldsymbol{\gamma}), \phi\} \\ &= \ln\Gamma(\phi) - \ln\Gamma[\mu_i(\boldsymbol{\alpha}, \boldsymbol{\gamma})\phi] - \ln\Gamma[(1 - \mu_i(\boldsymbol{\alpha}, \boldsymbol{\gamma}))\phi]. \end{aligned} \tag{S8}$$

The score equations can be derived straightforwardly:

$$\mathbf{U}_i = [\frac{\partial l_i(\boldsymbol{\alpha}, \boldsymbol{\gamma}, \phi)}{\partial\boldsymbol{\alpha}}, \frac{\partial l_i(\boldsymbol{\alpha}, \boldsymbol{\gamma}, \phi)}{\partial\boldsymbol{\gamma}}, \frac{\partial l_i(\boldsymbol{\alpha}, \boldsymbol{\gamma}, \phi)}{\partial\phi}]^T,$$

where

$$
\begin{aligned}
\frac{\partial l_i(\boldsymbol{\alpha},\boldsymbol{\gamma},\phi)}{\partial\boldsymbol{\alpha}} &= -\frac{\exp(\boldsymbol{X}_i^T\boldsymbol{\alpha})}{1+\exp(\boldsymbol{X}_i^T\boldsymbol{\alpha})}\boldsymbol{X}_i^T \\
&+ 1(y_i>0)\left[\boldsymbol{X}_i^T + h'_\alpha(\boldsymbol{\alpha},\boldsymbol{\gamma},\phi) - \frac{\exp(-\boldsymbol{X}_i^T\boldsymbol{\alpha})}{1+\exp(-\boldsymbol{X}_i^T\boldsymbol{\alpha})}\phi\,\mathrm{logit}(y_i)\boldsymbol{X}_i^T\right], \\
\frac{\partial l_i(\boldsymbol{\alpha},\boldsymbol{\gamma},\phi)}{\partial\boldsymbol{\gamma}} &= 1(y_i>0)\left[h'_\gamma(\boldsymbol{\alpha},\boldsymbol{\gamma},\phi) + \frac{\exp(\boldsymbol{X}_i^T\boldsymbol{\gamma})+\exp(\boldsymbol{X}_i^T\boldsymbol{\gamma}-\boldsymbol{X}_i^T\boldsymbol{\alpha})}{(1+\exp(\boldsymbol{X}_i^T\boldsymbol{\gamma}))^2}\phi\,\mathrm{logit}(y_i)\boldsymbol{X}_i^T\right], \\
\frac{\partial l_i(\boldsymbol{\alpha},\boldsymbol{\gamma},\phi)}{\partial\phi} &= 1(y_i>0)\left[h'_\phi(\boldsymbol{\alpha},\boldsymbol{\gamma},\phi) + \frac{1+\exp(-\boldsymbol{X}_i^T\boldsymbol{\alpha})}{1+\exp(-\boldsymbol{X}_i^T\boldsymbol{\gamma})}\mathrm{logit}(y_i) + \ln(1-y_i)\right],
\end{aligned}
$$

and

$$
\begin{aligned}
h'_\alpha(\boldsymbol{\alpha},\boldsymbol{\gamma},\phi) &= \frac{\mu'_{i\alpha}(\boldsymbol{\alpha},\boldsymbol{\gamma})}{\mu_i(\boldsymbol{\alpha},\boldsymbol{\gamma})} - \frac{\mu'_{i\alpha}(\boldsymbol{\alpha},\boldsymbol{\gamma})}{1-\mu_i(\boldsymbol{\alpha},\boldsymbol{\gamma})} \\
&+ \sum_{k=1}^{\infty}\left\{\frac{1}{k+\mu_i(\boldsymbol{\alpha},\boldsymbol{\gamma})\phi} - \frac{1}{k+[1-\mu_i(\boldsymbol{\alpha},\boldsymbol{\gamma})]\phi}\right\}\phi\mu'_{i\alpha}(\boldsymbol{\alpha},\boldsymbol{\gamma}), \\
h'_\gamma(\boldsymbol{\alpha},\boldsymbol{\gamma},\phi) &= \frac{\mu'_{i\gamma}(\boldsymbol{\alpha},\boldsymbol{\gamma})}{\mu_i(\boldsymbol{\alpha},\boldsymbol{\gamma})} - \frac{\mu'_{i\gamma}(\boldsymbol{\alpha},\boldsymbol{\gamma})}{1-\mu_i(\boldsymbol{\alpha},\boldsymbol{\gamma})} \\
&+ \sum_{k=1}^{\infty}\left\{\frac{1}{k+\mu_i(\boldsymbol{\alpha},\boldsymbol{\gamma})\phi} - \frac{1}{k+[1-\mu_i(\boldsymbol{\alpha},\boldsymbol{\gamma})]\phi}\right\}\phi\mu'_{i\gamma}(\boldsymbol{\alpha},\boldsymbol{\gamma}), \\
h'_\phi(\boldsymbol{\alpha},\boldsymbol{\gamma},\phi) &= \frac{1}{\phi} + \sum_{k=1}^{\infty}\left\{\frac{\mu_i(\boldsymbol{\alpha},\boldsymbol{\gamma})}{k+\mu_i(\boldsymbol{\alpha},\boldsymbol{\gamma})\phi} + \frac{1-\mu_i(\boldsymbol{\alpha},\boldsymbol{\gamma})}{k+(1-\mu_i(\boldsymbol{\alpha},\boldsymbol{\gamma}))\phi} - \frac{1}{k+\phi}\right\}; \\
\mu'_{i\alpha}(\boldsymbol{\alpha},\boldsymbol{\gamma}) &= -\frac{\exp(-\boldsymbol{X}_i^T\boldsymbol{\alpha})}{1+\exp(-\boldsymbol{X}_i^T\boldsymbol{\gamma})}\boldsymbol{X}_i^T, \\
\mu'_{i\gamma}(\boldsymbol{\alpha},\boldsymbol{\gamma}) &= \frac{\exp(\boldsymbol{X}_i^T\boldsymbol{\gamma})+\exp(\boldsymbol{X}_i^T\boldsymbol{\alpha})}{[1+\exp(\boldsymbol{X}_i^T\boldsymbol{\gamma})]^2}\boldsymbol{X}_i^T.
\end{aligned}
$$
