# Supplementary material for: A marginalized two-part Beta regression model for microbiome compositional data
Source: PLoS Comput Biol. 2018 Jul 23;14(7):e1006329. doi: 10.1371/journal.pcbi.1006329 (PMC6072097; doi:10.1371/journal.pcbi.1006329)
Supplement: S1 Code — The main SAS codes for the conventional two-part model and the proposed marginalized two-part model are shown in this section. (PDF) [file pcbi.1006329.s002.pdf]

S1 Code: The main SAS Codes for the two-part model.

In this part, we show SAS codes for the conventional two-part model and the proposed marginalized two-part model.

A: SAS code for the conventional two-part model

```
proc nlmixed data=one;

/* parameters and initial values;*/
parms alpha0=1.5 alpha1=1
      beta0=-2.5 beta1=1
      phi = 1;
bounds phi > 0;

/* model for the binary part;*/
temp1 = alpha0 + alpha1 * x1;
p = exp(temp1) / (1 + exp(temp1));

/* set up the likelihood;*/
if y = 0 then loglik = log(1 - p);
if y > 0 then do;
        temp2 = beta0 + beta1 * x1;
        mu = 1 / (1 + exp(-temp2));
        loglik = log(p) + lgamma(phi) - lgamma(mu * phi)
                 - lgamma((1 - mu) * phi) + (mu * phi - 1) * log(y)
                 + ((1 - mu) * phi - 1) * log(1 - y);
end;

/* fit model;*/
model y ~ general(loglik);

run;
```

B: SAS code for the marginalized two-part model

```
proc nlmixed data = one;

/* parameters and initial values;*/
parms alpha0=1.5 alpha1=1
      gamma0=-2.5 gamma1=1
      phi = 1;
bounds phi > 0;

/* model for the binary part;*/
temp1 = alpha0 + alpha1 * x1;
p = exp(temp1) / (1 + exp(temp1));

/* set up the likelihood;*/
if y = 0 then loglik = log(1 - p);
if y > 0 then do;
        temp2 = gamma0 + gamma1 * x1;
        mu = (1 + exp(-temp1)) / (1 + exp(-temp2));
        loglik = log(p) + lgamma(phi) - lgamma(mu * phi)
                 - lgamma((1 - mu) * phi) + (mu * phi - 1) * log(y)
                 + ((1 - mu) * phi - 1) * log(1 - y);
end;

/* fit model;*/
model y ~ general(loglik);

run;
```
