# Supplementary figures and images for: A marginalized two-part Beta regression model for microbiome compositional data
Source: PLoS Comput Biol. 2018 Jul 23;14(7):e1006329. doi: 10.1371/journal.pcbi.1006329 (PMC6072097; doi:10.1371/journal.pcbi.1006329)

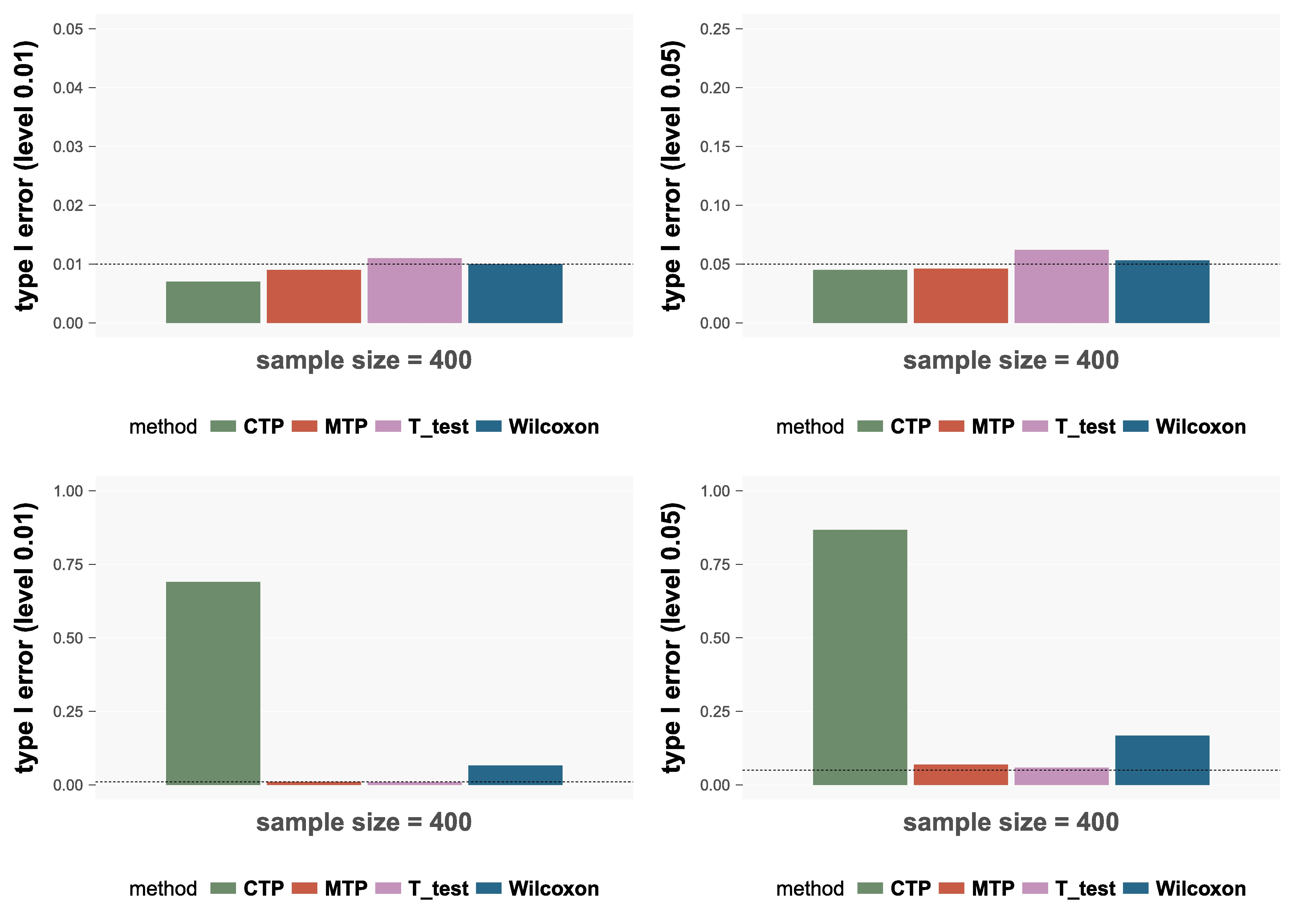

Supplement: S1 Fig — This figure shows the type I errors of the four methods for sample size 400. The results in the upper panels correspond to the setting α1 = 0, γ1 = 0 and the lower panels correspond to setting α1 = 1, γ1 = 0. In each setting, the left panel shows the results for significance level 0.01 and the right panel shows the results for significance level 0.05. The dashed horizontal line in each panel represents the significance level. (TIF) [file pcbi.1006329.s003.tif]

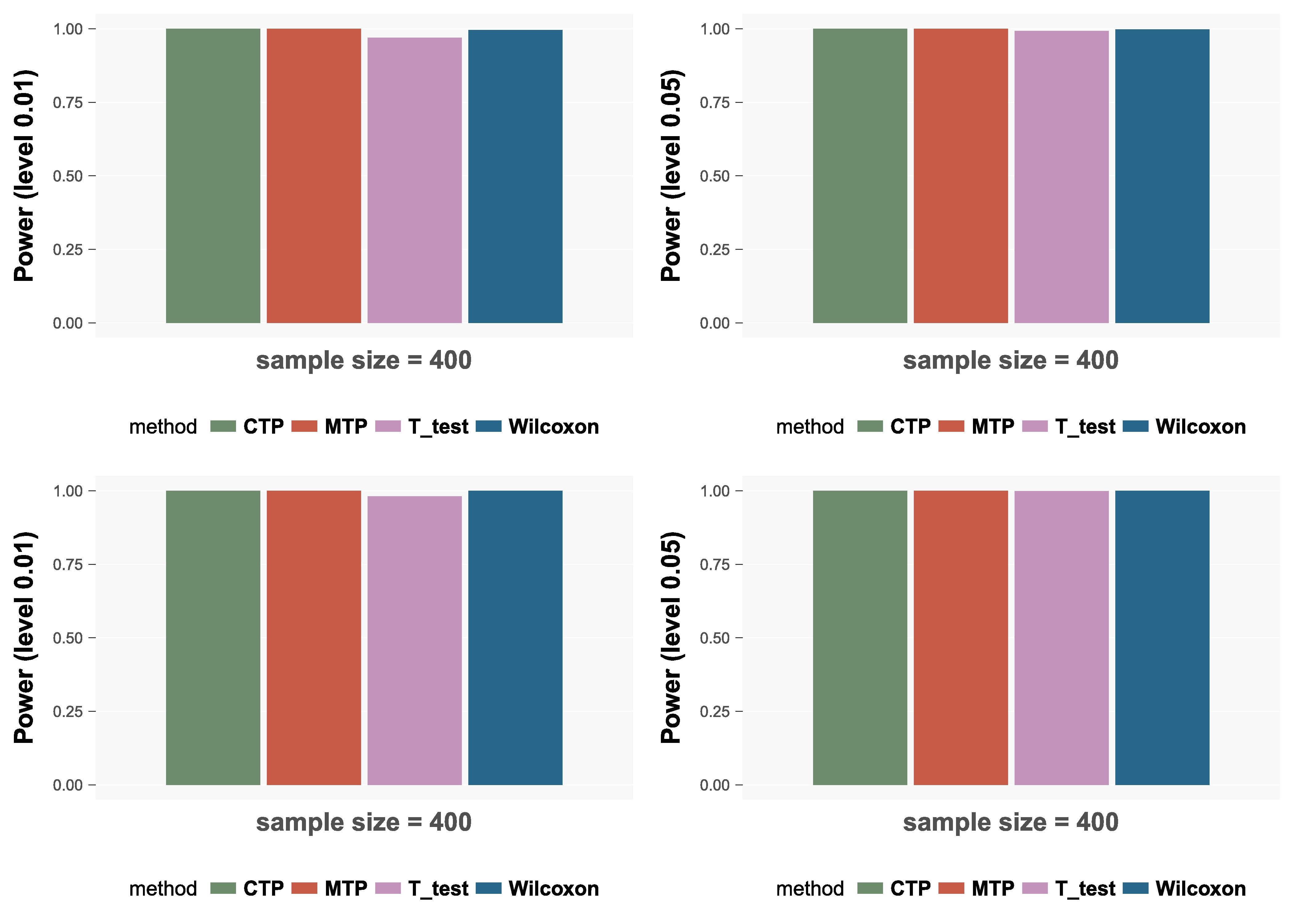

Supplement: S2 Fig — This figure shows the powers of the four methods for sample size 400. The upper panel contains the power corresponding to the setting α1 = 0, γ1 = 1 and the lower panel shows the power corresponding to the setting α1 = 1, γ1 = 1. In each setting, the left figure shows the results for significance level and the right panel shows the results for significance level 0.05. (TIF) [file pcbi.1006329.s004.tif]

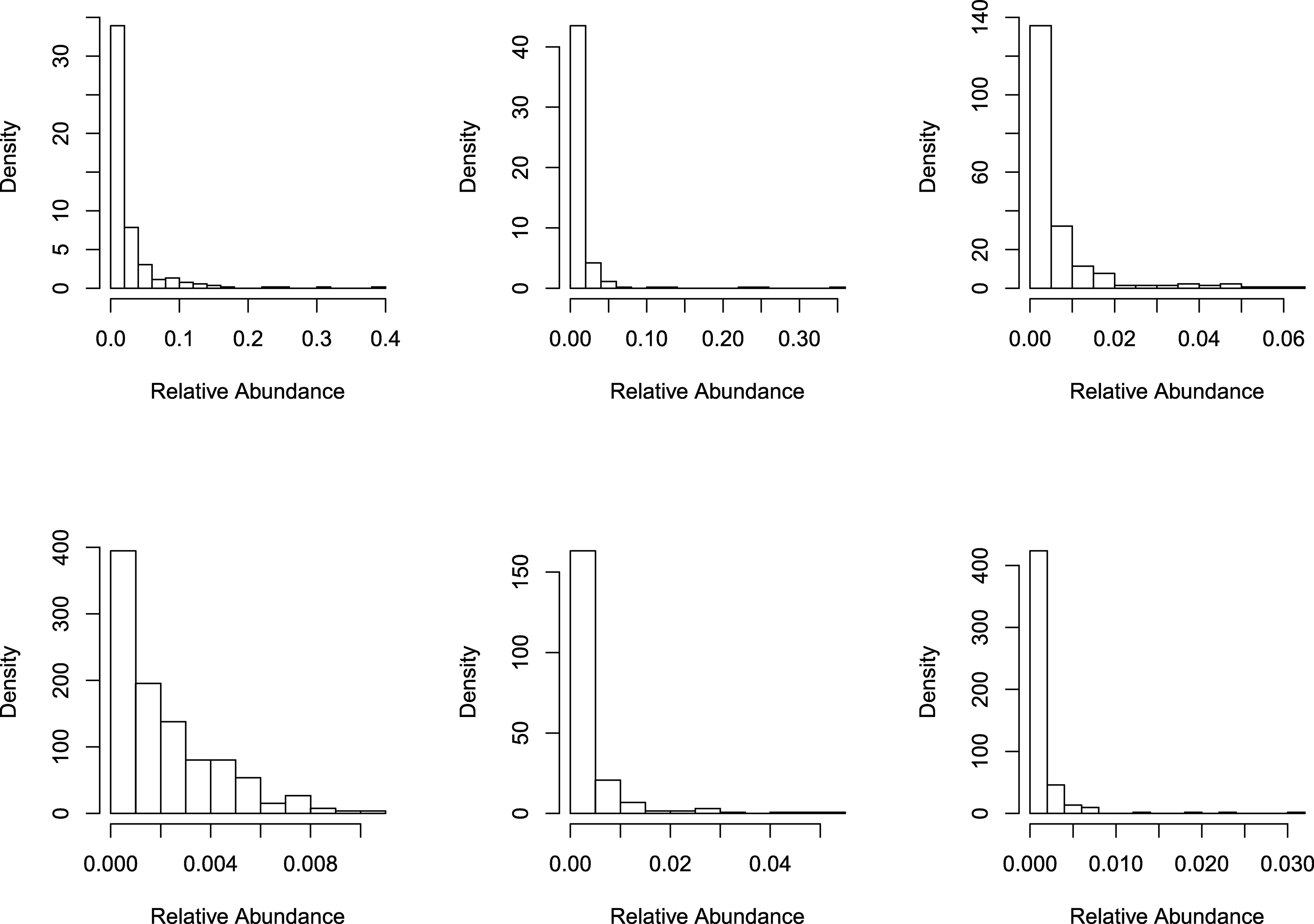

Supplement: S3 Fig — The figure shows the distributions of relative abundances of 6 OTUs. From the upper panel to the lower panel and from the left to the right, the proportions of zero values for these 6 OTUs are 0.77%, 3.45%, 4.97%, 14.18%, 29.89%, and 48.28%, respectively. (TIF) [file pcbi.1006329.s005.tif]

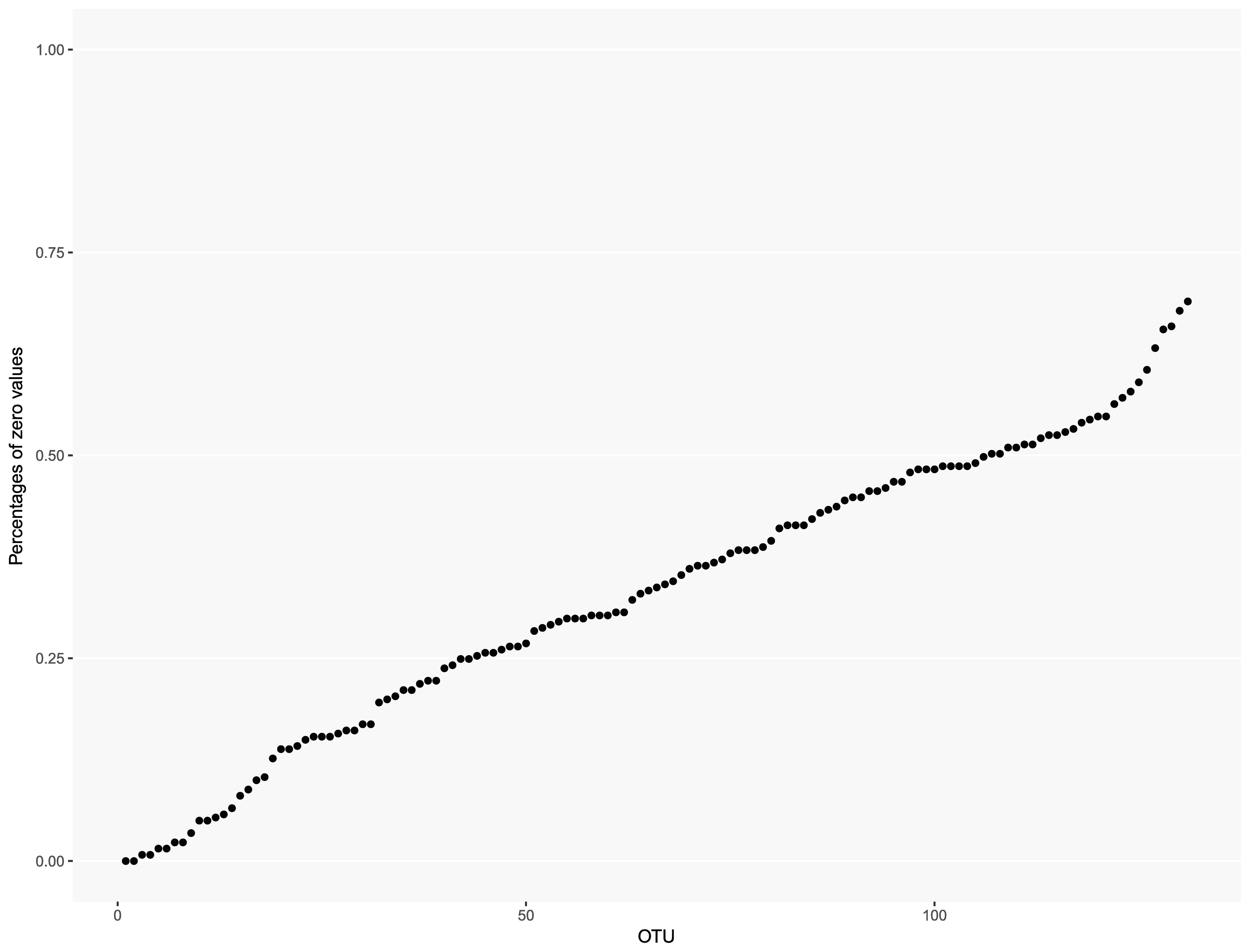

Supplement: S4 Fig — The lower quartile and the upper quartile of the percentages are 20.11% and 48.28%, respectively. (TIF) [file pcbi.1006329.s006.tif]

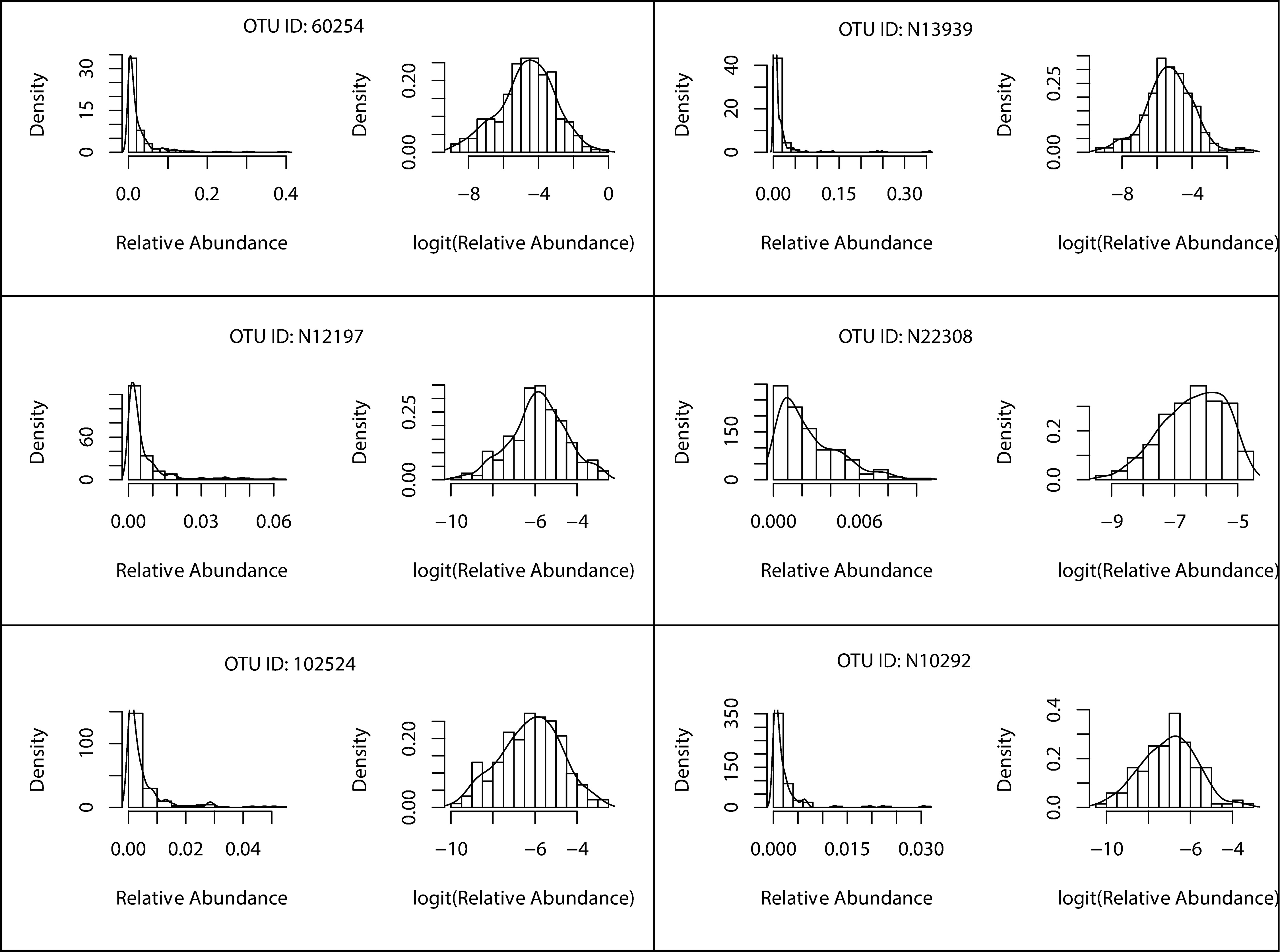

Supplement: S5 Fig — The figure shows the histogram of the relative abundance for 6 OTUs. The first one in every panel is the histogram of the OTU in the original scale, while the second one in every panel shows the histogram after logit transformation. (TIF) [file pcbi.1006329.s007.tif]
