# Supplementary material for: A marginalized two-part Beta regression model for microbiome compositional data
Source: PLoS Comput Biol. 2018 Jul 23;14(7):e1006329. doi: 10.1371/journal.pcbi.1006329 (PMC6072097; doi:10.1371/journal.pcbi.1006329)
Supplement: S1 Table — The table shows the detailed results of all the 45 OTUs that are identified by the proposed MTP model. (PDF) [file pcbi.1006329.s008.pdf]

S1 Table: The detailed results of the MTP model.

Table 1 shows all the 45 OTUs identified by the proposed MTP model. As in [17], for OTUs which cannot be classified at the species level, the next highest classifiable taxonomic level (denoted by 'o', 'f' and 'g' for order, family and genus) is displayed. We use a number in the superscript to distinguish among different OTUs with the same classification name. The OTUs are ranked by their p Values. Est and SE are the the estimation and standard error of the coefficient of treatment γ in Model (4).

Table 1: All 45 OTUs identified by the MTP model.

Rank	ID	Species	Est	SE	p Value
1	237040	g_Alicyclobacillus	2.3262	0.3148	$< 1E-16$
2	101810	g_Helicobacter^1	-1.3934	0.1460	$< 1E-16$
3	52884	g_Helicobacter^2	1.9091	0.2875	4.88E-15
4	N10167	o_Bacteroidales^1	2.5761	0.4234	8.33E-15
5	381715	f_Ruminococcaceae	2.7674	0.4777	6.82E-14
6	269548	g_Helicobacter^3	1.2357	0.1833	3.57E-13
7	N26397	Acetobacter aceti	1.8841	0.3055	4.86E-13
8	294146	Acetobacterorleanensis	2.0662	0.3387	5.66E-13
9	N2007	Acinetobacterlwoffii	2.5549	0.4498	1.19E-12
10	N8891	g_Mucispirillum	1.5868	0.2679	1.53E-11
11	591285	g_Bifidobacterium	2.3502	0.4366	1.17E-10
12	108747	g_Streptococcus^1	1.0750	0.1757	1.44E-10
13	15711	Bacteroides acidifaciens	2.6413	0.5146	2.12E-10
14	N13471	Bacteroides uniformis	1.3055	0.2247	2.36E-10
15	157775	Bifidobacterium longum	1.6902	0.3125	1.03E-09
16	N19020	Clostridium cocleatum	1.0886	0.1900	1.79E-09
17	263010	f_Flavobacteriaceae	-1.2452	0.1915	3.33E-09
18	252321	g_Lactobacillus^1	1.8870	0.3655	4.68E-09
19	173469	Helicobacter apodemus	0.8340	0.1456	5.71E-09
20	N10292	Helicobacter ganmani	1.9825	0.4006	9.59E-09
21	182382	Helicobacter hepaticus	1.2208	0.2346	1.93E-08
22	N4396	g_Acetobacter^1	-1.4977	0.2664	1.65E-07
23	N24810	Lactobacillus helveticus	1.1301	0.2293	1.94E-07
24	N5309	Mucispirillum schaedleri	1.1098	0.2284	3.31E-07
25	538741	g_Staphylococcus^1	0.8332	0.1758	1.57E-06
26	N79	o_Bacteroidales^2	1.6492	0.3745	1.79E-06
27	N10650	g_Corynebacterium	-0.9877	0.1893	4.12E-06
28	567604	g_Lactobacillus^2	1.0297	0.2320	4.36E-06
29	264534	o_Bacteroidales^3	1.1382	0.2649	6.46E-06
30	331878	f_Lachnospiraceae	0.7426	0.1626	6.70E-06
31	66816	g_Pseudomonas	0.8344	0.1869	6.89E-06
32	587004	Staphylococcus aureus	-0.9661	0.1959	7.61E-06
33	288912	Staphylococcus hominis^1	-0.8412	0.1719	1.74E-05
34	356164	Staphylococcus hominis^2	1.0520	0.2535	1.87E-05

Table 1: All 45 OTUs identified by the MTP model.

Rank	ID	Species	Est	SE	p Value
35	N29011	g_Staphylococcus[2]	0.6584	0.1497	2.30E-05
36	N20648	Streptococcus thermophilus	-1.2108	0.2547	2.41E-05
37	308167	Streptococcussuis	1.4801	0.3669	2.57E-05
38	319794	o_Bacteroidales[4]	1.1850	0.2872	2.94E-05
39	N31208	g_Streptococcus[2]	0.6256	0.1513	5.51E-05
40	N19525	g_Staphylococcus[3]	0.6644	0.1612	5.54E-05
41	35624	g_Streptococcus[3]	-1.2305	0.2718	6.59E-05
42	549374	g_Acetobacter[2]	-1.0425	0.2490	1.77E-04
43	469832	g_Bacteroides	0.7012	0.1846	2.25E-04
44	314572	g_Acinetobacter	-0.6442	0.1511	2.35E-04
45	268155	o_Bacteroidales[5]	0.6134	0.1627	3.25E-04
